# Personality-dependent breeding dispersal in rural but not urban burrowing owls

**DOI:** 10.1038/s41598-019-39251-w

**Published:** 2019-02-27

**Authors:** Álvaro Luna, Antonio Palma, Ana Sanz-Aguilar, José L. Tella, Martina Carrete

**Affiliations:** 1 Department of Conservation Biology, Estación Biológica de Doñana - CSIC, Sevilla, Spain; 2 Population Ecology Group, Institut Mediterrani d’Estudis Avançats IMEDEA (CSIC-UIB), Mallorca, Spain; 3 Department of Physical, Chemical and Natural Systems, Universidad Pablo de Olavide, Sevilla, Spain

**Keywords:** Behavioural ecology, Urban ecology

## Abstract

Dispersal propensity has been correlated with personality traits, conspecific density and predation risk in a variety of species. Thus, changes in the relative frequency of behavioural phenotypes or in the ecological pressures faced by individuals in contrasting habitats can have unexpected effects on their dispersal strategies. Here, using the burrowing owl *Athene cunicularia* as a study model, we test whether changes in the behavioural profile of individuals and changes in conspecific density and predation pressure associated with urban life influence their breeding dispersal decisions compared to rural conspecifics. Our results show that breeding dispersal behaviour differs between rural and urban individuals. Site fidelity was lower among rural than among urban birds, and primarily related to an individual’s behaviours (fear of humans), which has been reported to reflect individual personality. In contrast, the main determinant of site fidelity among urban owls was conspecific density. After taking the decision of dispersing, urban owls moved shorter distances than rural ones, with females dispersing farther than males. Our results support a personality-dependent dispersal pattern that might vary with predation risk. However, as multiple individuals of two populations (one urban, one rural) were used for this research, differences can thus also be caused by other factors differing between the two populations. Further research is needed to properly understand the ecological and evolutionary consequences of changes in dispersal behaviours, especially in terms of population structuring and gene flow between urban and rural populations.

## Introduction

Dispersal has important consequences for an individual’s fitness, affecting population structure, species distributions, and range shifts^1,2^. Thus, understanding why and how organisms disperse is central to many questions in theoretical and applied ecology and evolution^3,4^. Breeding dispersal (i.e. the inter-annual movement of individuals between breeding sites) has received much attention in recent decades, especially for birds. Within this taxonomic group, breeding dispersal has been related to an individual’s characteristics such as age or sex, suggesting differences in gender roles in territory acquisition^1^ as well as benefits derived from breeding-site familiarity^3,5,6^. However, within a sex or age class, individual variation in dispersal movements can be important and linked to an individual’s experience such as breeding success, mate loss, or predation pressure in the previous year^2,7,8^, which can be ultimately influenced by habitat quality^9^ and modulated by conspecific density^10^. A recent theory has suggested that individual variation in dispersal may also be linked to individual differences in behavioural types or behavioural syndromes that can be stable over the ontogeny or across situations (defined as a given set of conditions at one point in time, involving different levels along an environmental gradient or different sets of conditions across time)^11^, leading a personality-dependent dispersal^12,13^. However, as behavioural differences between individuals usually influence their vulnerability to predation^14,15^ the pattern of personality-dependent dispersal can be modified when factors motivating dispersal, such as predation risk, change^16^. Thus, studies contrasting dispersal patterns of conspecifics subjected to different selection regimes can help us better understand the dynamic nature of dispersal as well as its drivers. This is particularly important in the context of global change, as dispersal is a crucial mechanism allowing species to respond to shifting environmental conditions^8^.

Urbanisation is one of the most prevailing causes of habitat transformation worldwide and a main driver of global change. Although animal communities are usually simplified and homogenised in these new habitats^17^, cities can also act as predator-free refuges for the many species able to colonise them^18^. This colonisation of urban environments by birds has been related to their inter-individual variability in fear of humans (a repeatable and heritable behaviour which is correlated with exploration and antipredatory behaviour and can be considered as a personality trait)^19,20^, such that urban life would select for fearless individuals^21,22^. In urban areas, these individuals can improve their demographic parameters, as predation risk is lower, and establish larger population densities than in rural habitats, even changing the habitat selection pattern of a species^23,24^. However, due to the role of behaviour, conspecific density and predation pressure on the dispersal propensity of individuals, urbanisation can deeply affect not only the demography but also the spatial structure and dynamics of rural and urban populations. In spite of this, studies comparing breeding dispersal behaviour of individuals living in both habitat types are scarce^25^ and no one has deeply explored the mechanism provoking these differences.

Here, using the burrowing owl *Athene cunicularia* as a study model, we assessed the importance of an individual’s traits (age, sex, and behaviour), previous breeding experiences (breeding output and nest predation) and conspecific density in determining individual breeding dispersal behaviour in rural and urban birds. We specifically considered how an individual’s personality affects site fidelity and breeding dispersal distances, discussing which changes associated with urban life (i.e. reduction in predation pressure, increments in conspecific density or selection of individuals tolerant towards humans) can explain differences in the dispersal patterns of rural and urban birds. Our results showed that rural birds were less faithful to their breeding territories and dispersed at greater distances than urban ones. Our findings support the personality-dependent dispersal hypothesis and a role for the behavioural skewness associated with urban invasion in explaining changes in dispersal patterns of urban and rural birds, highlighting the potential of urbanisation to cause population structuring by altering individual’s movements.

## Results

**Figure 1:**
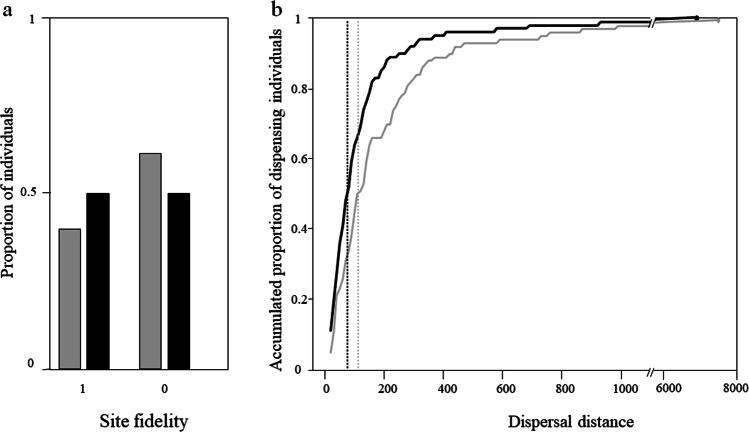
(**a**) Proportion of burrowing owls showing site fidelity (1) or changing their breeding sites between successive years (0) in rural (grey bars) and urban (black bars) habitats. (**b**) For individuals changing their breeding sites, the accumulated proportion of dispersing urban (grey line) and rural (black line) individuals as a funciton of distance is also shown. The maximum dispersal distance observed is indicated by a point (grey and black, for urban and rural birds respectively). Vertical dashed lines show mean distances for urban (grey line) and rural (black line) birds.

We recorded 866 breeding dispersal events in 130 rural and 334 urban owls. Rural birds were less faithful to their breeding sites (rural: 39%, urban: 50%; χ^2^ = 6.23, d.f. = 1, p = 0.0126; Fig. 1a) and, when they dispersed, did so at greater distances than urban birds (median rural: 112 m, range: 13–7,500 m, median urban: 76.5 m, range: 11–6,900 m; F_1,348_ = 40.67, p < 0.0001; Fig. 1b).

**Figure 2:**
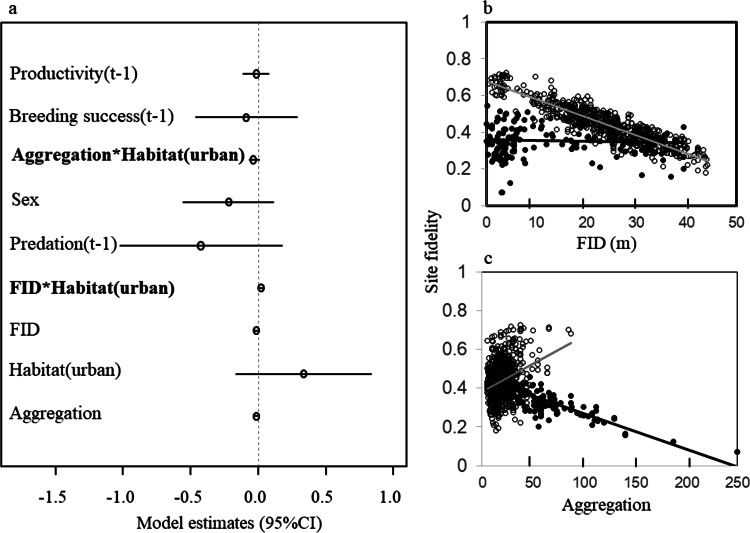
(**a**) Factors affecting site fidelity among rural and urban burrowing owls (estimate ± 95% CI). Site fidelity was negatively related to individual behaviour (measured as flight initiation distances, FID) among rural individuals (**b**) while it was negatively related to conspecific density (measured as aggregation) among urban ones. (**c**) Lines (black: rural, grey: urban) show the probability of remaining in the same breeding site for individuals with different FID and living at different conspecific densities. Dots (black: rural, white: urban) show predicted values.

Our reduced dataset of birds ringed as chicks show that age was not related to site fidelity or dispersal distance among urban and rural birds (Tables 1 and S1). Thus, we relied on our larger sample including all individuals to assess the relative importance of an individual’s traits, previous experience and conspecific density on the dispersal pattern of individuals. Using this dataset, we obtained alternative models including individual traits as well as descriptors of previous experience (Table 2). After model averaging, we found strong support for an effect of individual behaviour on site fidelity of rural birds and of conspecific density on site fidelity of urban and rural ones (Table 2 and Fig. 2), shyer rural individuals and birds breeding at higher conspecific densities having a higher probability of changing their breeding sites between successive years than their counterparts (R^2^ = 0.16). Habitat, and breeding success and productivity in the previous year received strong support to explain variability in the dispersal distance of all individuals (R^2^ = 0.46), urban birds, and individuals breeding successfully or having more chicks moving less than rural, and unsuccessful owls (Table 2 and Fig. 2). Models obtained using AICc arose similar results, also supporting a role for conspecific aggregation in site fidelity of urban birds and sex on dispersal distance (females dispersing further than males; Table S2).

**Table 1:** Alternative models (∆BIC < 6) obtained to assess the relative importance of individual’s traits (age, sex and behaviour, measured as FID), previous breeding experience (breeding success, productivity and predation in the previous year *t* − 1) and conspecific density on the dispersal pattern (site fidelity and dispersal distances) of rural and urban (habitat) burrowing owls *Athene cunicularia*.

	df	BIC	ΔBIC	weight
**Site fidelity**				
Null	3	211.19	0.00	0.36
Productivity (t − 1)	4	213.96	2.78	0.09
Predation (t − 1)	4	214.13	2.94	0.08
Breeding success (t − 1)	4	214.31	3.13	0.08
Habitat	4	215.31	4.13	0.05
Sex	4	215.55	4.36	0.04
Aggregation	4	216.07	4.89	0.03
FID	4	216.16	4.97	0.03
Habitat * FID	5	216.40	5.22	0.03
**Dispersal distance**				
Null	4	75.44	0.00	0.68
Habitat	5	78.25	2.81	0.17

Models were run using information from individuals of known age (ringed as chicks). See Table S1 for alternative models obtained using the Akaike Information Criterion corrected for small sample sizes (AICc).

**Table 2:** Relative importance of an individual’s traits (sex and behaviour, measured as FID), previous breeding experience (breeding success, productivity and predation in the previous year *t* − 1) and conspecific density on the dispersal pattern (site fidelity and dispersal distances) of rural and urban (habitat) burrowing owls *Athene cunicularia*.

	df	BIC	ΔBIC	weight	Variables	Estimate	2.5%	97.5%
**Site fidelity**								
FID	4	1083.00	0.00	0.30	**FID**	−**0**.**01**	−**0**.**02**	**0**
Habitat	4	1084.18	1.18	0.17	**Habitat (urban)**	**0**.**51**	**0**.**08**	**0**.**94**
Null	3	1084.69	1.69	0.13	**FID** * **Habitat (rural)**	−**0**.**02**	−**0**.**03**	−**0**.**01**
Habitat * FID	5	1085.70	2.70	0.08	FID * Habitat (urban)	0	−0.01	0.02
FID, Predation (t − 1)	5	1087.57	4.57	0.03	Predation (t − 1)	−0.44	−1.04	0.16
Aggregation + FID	5	1087.81	4.81	0.03	**Aggregation**	−**0**.**01**	−**0**.**03**	**0**
Habitat, FID	5	1088.03	5.03	0.02	Sex (female)	−0.24	−0.57	0.09
Sex, FID	5	1088.15	5.15	0.02				
Sex	4	1088.46	5.47	0.02				
Habitat, Sex	5	1088.76	5.76	0.02				
Habitat, Aggregation	5	1088.84	5.84	0.02				
**Dispersal distance**								
Habitat, Breeding success (t − 1)	6	471.18	0.00	0.53	**Habitat(urban)**	−**0**.**22**	−**0**.**37**	−**0**.**07**
Habitat	5	472.78	1.61	0.24	**Breeding success (t** **−** **1)**	−**0**.**19**	−**0**.**35**	−**0**.**04**
Breeding success (t − 1)	5	476.05	4.88	0.05	Breeding success (t − 1) * Habitat(urban)	0.21	−0.01	0.43
Habitat * Breeding success (t − 1)	7	476.07	4.90	0.05	**Productivity (t** **−** **1)**	−**0**.**04**	−**0**.**06**	−**0**.**01**
Habitat * Breeding success (t − 1)	7	476.07	4.90	0.05				
Habitat, Productivity (t − 1)	6	477.05	5.88	0.03				

Estimates and 95% confidence intervals (2.5% and 97.5%) were assessed after model averaging. We considered that a given variable has no, weak or strong support when the 95% confidence interval strongly overlapped zero, barely overlapped zero, or did not overlap zero (in bold), respectively. Models were run using all individuals of unknown age, as age has not received statistical support (see Tables 1 and S1). Models shown are those used for model averaging (ΔBIC ≤ 6). See Table S2 for results obtained using the Akaike Information Criterion (ΔAICc < 6).

The lack of support for individual behaviour explaining site fidelity among urban birds could be a consequence of the reduced variability shown by this variable compared to values observed among rural individuals (range FID: rural: 5–250 m, urban: 5–87 m). However, when models were run using a subset of rural individuals with FIDs within the range of urban birds, the results remained similar, and rural, shy individuals were still less faithful to their breeding sites than bolder (Table 3; R^2^ = 0.10). Accordingly, individuals breeding successfully also dispersed at lower distances than unsuccessful ones (Tables 3 and S3; R^2^ = 0.57).

**Table 3:** Relative importance of an individual’s traits (sex and behaviour, measured as FID), previous breeding experience (breeding success, productivity and predation in the previous year *t* − 1) and conspecific density on the dispersal pattern (site fidelity and dispersal distances) of rural burrowing owls *Athene cunicularia* with FID within the range of urban ones (5–87 m).

	df	BIC	ΔBIC	weight	Variables	Estimate	2.5%	97.5%
**Site fidelity**								
FID	4	210.05	0.00	0.29	**FID**	−**0**.**39**	−**0**.**76**	−**0**.**02**
Null	3	210.09	0.03	0.29	Sex2	−0.39	−1.11	0.32
Sex	4	213.36	3.30	0.06	Productivity (t − 1)	−0.15	−0.5	0.19
FID, Productivity (t − 1)	5	214.23	4.18	0.04	Breeding success (t − 1)	−0.34	−1.1	0.42
Breeding success (t − 1)	4	214.24	4.19	0.04	Aggregation	0.12	−0.22	0.46
FID, Breeding success (t − 1)	5	214.27	4.22	0.04	Predation (t − 1)	−0.42	−1.57	0.72
Productivity (t − 1)	4	214.29	4.24	0.03				
Sex, FID	5	214.41	4.35	0.03				
Aggregation	4	214.43	4.37	0.03				
Predation (t − 1)	4	214.48	4.43	0.03				
FID, Predation (t − 1)	5	214.50	4.45	0.03				
Aggregation, FID	5	214.73	4.67	0.03				
**Dispersal distance**								
Breeding success (t − 1)	5	114.18	0.00	0.92	Breeding success (t − 1)	−0.40	−0.60	−0.20

Estimates and 95% confidence intervals (2.5% and 97.5%) were assessed after model averaging. We considered that a given variable has no, weak or strong support when the 95% confidence interval strongly overlapped zero, barely overlapped zero, or did not overlap zero (in bold), respectively. Models shown are those used for model averaging (ΔBIC ≤ 6). See Table S3 for results obtained using the Akaike Information Criterion (ΔAICc < 6).

## Discussion

Our results show that determinants of breeding dispersal differ between rural and urban burrowing owls. Site fidelity among rural birds was lower than among urban ones, and was primarily related to individual behaviour (measured here as fear of humans), such that shy individuals were more prone to changing their breeding sites between successive years than bold ones. This result challenges previous studies showing that emigrants, immigrants or colonizers are usually bolder, more exploratory, or aggressive than residents or locally born individuals^26,27^. A potential explanation for this difference could be that, in our population, shy rural birds are also less aggressive toward predators^22^, and may disperse from their breeding sites to avoid the abundant predators present in rural areas^8^. Previous studies have shown that high predation pressure explained the reduced breeding success and productivity of this rural population compared to the urban one^18^. Accordingly, we found a relationship between the experience of individuals in one year (breeding success and productivity) and their dispersal distance in the following year, so that unsuccessful individuals dispersed farther than successful ones. Contrary to rural birds, urban individuals were more faithful to their breeding sites and dispersed, in general, shorter distances during consecutive breeding seasons. Moreover, the main determinant of site fidelity among urban individuals was conspecific density, such that individuals breeding at higher aggregations have a higher probability of dispersing than those occupying sparser areas. Positive density-dependent dispersal has been previously described in other taxa^28,29^, and can arise due to competitive processes between the densely distributed urban pairs (ca. seven times higher than rural ones)^18^. However, no relationship between site fidelity or dispersal distance and individual behaviour was detected among urban owls. Although this result may be related to the low variability in FID within urban birds, when rural individuals with profiles similar to those of urban birds were considered (i.e. FID ranging within values observed among urban birds: 0–87 m), we still found a negative association between behaviour and site fidelity. This suggests more than a statistical issue, and that a personality-dependent dispersal pattern that varies between rural and urban habitats and is likely associated with predation risk is at play. Thus, in environments with high-predation risk (rural habitats), shy individuals, unlike bold ones, would be strongly limited in the number of suitable habitats (in terms of predation risk) they may occupy, thereby making behavioural differences between the two types more pronounced. On the contrary, predator release in urban environments would exert no constraints on individual movements, whatever their tendency to take risks, hence cancelling the personality-dependent dispersal pattern observed among rural individuals. Alternatively, or complementarily, it is also possible that the selection of bolder individuals during urban invasion could be dismantling the effect of personality on dispersal among urban individuals, as the absence of correlation between antipredatory behaviour and fear of humans among urban birds^22^ could be reducing the relationship between fear of humans and dispersal in these habitats. It is worth mentioning that these results could have arisen as a consequence of differences in the resighting probability of birds related to personality and habitat type so that the covariance found in rural areas might represent an artefact caused by personality-related detection bias. However, our recapture probability in the study area is very high and not related to FID (see Supplementary materials).

Recent papers have explored how personality-dependent dispersal is affected in varying environments^13^, and its importance in spatial ecology and some global change scenarios, in particular, biological invasions and habitat fragmentation^30,31^. Although urbanisation represents one of the most prevailing causes of habitat transformation worldwide and despite its profound effects on demography and behaviour^25,32^, there are no studies dealing with its potential role in changing the dispersal patterns of individuals. At an interspecific level, previous studies have suggested a relationship between FID and descriptors of natal dispersal or range distribution^33,34^. Here, we provide the first evidence, at the individual level, that breeding dispersal is also personality-dependent in a bird species, suggesting that changes in ecological conditions associated with urban life (increments in breeding success mainly through predation release, but also the selection of human-tolerant phenotypes) can dismantle this relationship, favouring site fidelity and short dispersal movements. This change in breeding dispersal behaviour is likely contributing to the genetic structure detected between three different urban and one panmictic rural populations of burrowing owls, but also between urban cores separated by a few kilometres^35^.

Our study has been performed using information on breeding dispersal at the individual level, controlling for the lack of independence in these data by including individual as a random term. These individuals, which belong to two separate groups (urban and rural), were compared and differences discussed in the context of changes associated to urban life. It is true that our study has been performed in only one urban-rural pair of populations, which prevent us to emphatically assess that differences in the dispersal patterns of our study units are due to urbanization. However, the contrasted characteristics of both habitats are tightly linked to their degree of urbanization, and differences observed among individuals living in urban and rural areas across the world have shown similar patterns than those obtained here and which have been related to dispersal in the present study (i.e., the bolder behaviour of urban individuals^36–39^, the higher conspecific density of urban cores^40–43^, the higher breeding parameters of urban compared to rural populations^44^ or the colonization of urban areas from a pool of rural individuals)^39,45^. Thus, it is very likely that the dispersal patterns described here can be extended to other urban-rural areas. Further research, however, is needed to make stronger generalizations to properly understand the ecological and evolutionary consequences of these differences in dispersal between urban and rural birds and its implications for population structuring and gene flow between urban and rural populations.

## Material and Methods

### Study species and area

The burrowing owl is distributed across American open landscapes, breeding in burrows excavated by the owls themselves or by mammals. Breeding pairs are territorial and show diurnal activity, and are easily located in the surroundings of their nests^20^. In our study area (ca. 5,400 km^2^ of rural and urban areas around Bahia Blanca city, Argentina), rural owls breed in large extensions of natural grasslands and pastures dedicated to extensive livestock grazing and low-intensive cereal crops, where human presence is extremely low and mostly restricted to a few paved or unpaved roads^46^. Urban owls, conversely, excavate their nests in small private and public gardens in urbanised residential areas, unbuilt spaces among houses, curbs of streets and large avenues, and are in constant contact with homeowners, children, pedestrians and intense car traffic. The city is immediately surrounded by large and flat rural extensions, with no obstacles preventing owls from moving between habitats^19^. Moreover, as owls are able to excavate their own burrows, there are no habitat constraints (e.g., availability of nesting structures) that can limit their dispersal movements.

From 2006 to 2016, we annually monitored the breeding population of the species in the study area, totalling ca. 2,200 urban and 3,000 rural nests during the whole period. The location of all occupied nests was used to calculate an annual aggregation index for each breeding pair as their relative position within the spatial distribution of all breeding pairs^32^. This index, which reflects conspecific density, was obtained as *S*_*i*_ = *Σ exp* (−*d*_*ij*_) (with *i* ≠ *j*), where *d*_*ij*_ was the linear distance between pairs *i* and *j*. Territories were repeatedly visited to assess breeding success (i.e. breeding pairs successfully producing at least one fledgling) and productivity (i.e. the number of young fledged per breeding attempt), and to look for signs of predation such as the presence of corpses or plucked owl feathers at the entrance of the nests (see Rebolo-Ifrán *et al*.^18^ for a more detailed methodology). During this period, we also captured ca. 2,000 adults and chicks using bow nets and ribbon carpets to mark them with plastic colour-numbered rings readable at distance. Individuals were sexed based on plumage characteristics^19^ and, when needed, by molecular procedures^47^. Resightings of marked birds were done during an intensive population monitoring program lasting from 2007 to 2016, surveying all known breeding sites as well as unoccupied but suitable areas. Fear of humans is indicative of the risk that individuals are willing to take in our presence, and has been shown to be key to understanding avian urban invasion^23^. This behaviour is highly repeatable along an individual adulthood^19^, heritable^20^ and linked to exploration and antipredatory behaviours^22^, and is thus a consistent predictor of individual personality. We measured fear of humans of breeding birds during the chick-rearing period as the distance at which individuals flee when approached by a human (so-called flight initiation distance, hereafter FID), following standard protocols (see details in^18,20^). Both single and average values of FID were used (when more than one measure was obtained from a single individual in the same or different years), given the high repeatability of this behaviour^19^.

### Statistical approach

We used Generalized Linear Mixed Models (GLMM) to compare site fidelity (logistic link function, binomial error distribution) and dispersal distances (log-transformed, identity link function, normal error distribution) between rural and urban owls and to explore the effects of individual traits (sex, age, and FID), previous breeding experience (breeding success, productivity and predation), and conspecific density on these parameters. We considered that a bird remained faithful to its previous-year breeding site when it stayed in the same nest or in its immediate surroundings (radius = 10 m) between successive breeding events. This distance was established based on the GPS location error (3–8 m) and given that holes at distances ≤10 m can be different entrances to the same burrow. For individuals moving farther than 10 m (categorized as dispersers), we measured their dispersal distances as the straight-line between two consecutive breeding sites.

We assessed the relative contribution of individual traits, previous experience and conspecific density in determining the dispersal patterns of rural and urban burrowing owls using an information-theoretic approach on two main datasets. First, we performed models using the group of individuals of known age (captured as chicks in their nests), to explore the role of age in dispersal. Then, as most individuals were captured as adults and their age was unknown, we ran a second set of models without considering the effect of age. Models were built using a different combination of variables in interaction with habitat, but including alternatively only one descriptor of an individual’s previous experience due to their high correlational causation (predation is the main cause of breeding failure in the study species, thus affecting breeding success and productivity)^18^ and multicollinearity. All models included “individual” and “year” as random terms to control for pseudoreplication and potential interannual differences, respectively.

Model selection was performed using the Bayes Information Criterion, BIC^48^. Within each set of models (which includes the null model), we calculated the ΔBIC*i* (as the difference between the BIC of model *i* and that of the best model) and the weight (*w*)^34^ of each model. Models within 6 BIC units of the best one were considered as alternatives and used to perform model averaging (MuMIn package)^49^. BIC penalizes larger models more heavily than other criteria such as the Akaike Information Criteria (AIC) and so will tend to prefer smaller models sometimes losing some weak relationships. Thus, we also used the AICc (Akaike Information Criterion corrected for small sample sizes) to checked the consistency of our findings (results obtained using AICc are only shown in Supplementary Materials). All continuous variables were centred before modelling to properly estimate their main effects regardless of whether we include the interaction^50^. We considered that a given effect received no, weak or strong statistic support when the 95% confidence interval (CI) strongly overlapped zero, barely overlapped zero, or did not overlap zero, respectively. Complementarity, we calculated the coefficient of determination, R^2^, as a measure of the variance explained by a model^51^. Statistical analyses were conducted in R 3.1.2^52^.

### Ethics statements

Fieldwork and procedures were conducted under permits from the Argentinean wildlife agency (22500-4102/09), and the owners of private properties, in accordance with the approved guidelines of the Ethics Committee of CSIC (CEBA-EBD-11-28).

## Supplementary information

Supplementary_Info
