# Author Correction: Personality-dependent breeding dispersal in rural but not urban burrowing owls

**DOI:** 10.1038/s41598-020-58513-6

**Published:** 2020-01-29

**Authors:** Álvaro Luna, Antonio Palma, Ana Sanz-Aguilar, José L. Tella, Martina Carrete

**Affiliations:** 1 Department of Conservation Biology, Estación Biológica de Doñana - CSIC, Sevilla, Spain; 2 Population Ecology Group, Institut Mediterrani d’Estudis Avançats IMEDEA (CSIC-UIB), Mallorca, Spain; 3 Department of Physical, Chemical and Natural Systems, Universidad Pablo de Olavide, Sevilla, Spain

Correction to: *Scientific Reports* 10.1038/s41598-019-39251-w, published online 27 February 2019

This Article contains errors in the Results section, where:

“After model averaging, we found strong support for an effect of individual behaviour on site fidelity of rural birds and of conspecific density on site fidelity of urban and rural ones (Table 2 and Fig. 2), shyer rural individuals and birds breeding at higher conspecific densities having a higher probability of changing their breeding sites between successive years than their counterparts (R2 = 0.16). Habitat, and breeding success and productivity in the previous year received strong support to explain variability in the dispersal distance of all individuals (R2 = 0.46), urban birds, and individuals breeding successfully or having more chicks moving less than rural, and unsuccessful owls (Table 2 and Fig. 2).”

should read:

“After model averaging, we found strong support for an effect of individual behaviour on site fidelity of rural birds and of conspecific density on site fidelity of urban and rural ones (Table 2 and Fig. 2), shyer rural individuals and urban birds breeding at higher conspecific densities having a higher probability of changing their breeding sites between successive years than their counterparts (R2 = 0.16). Habitat, and breeding success and productivity in the previous year received strong support to explain variability in the dispersal distance of all individuals (R2 = 0.46), urban birds, and individuals breeding successfully or having more chicks moving less than rural, and unsuccessful owls (Table 2).”

Additionally, there is an error in the Legend of Figure 1, where:

“(a) Proportion of burrowing owls showing site fidelity (1) or changing their breeding sites between successive years (0) in rural (grey bars) and urban (black bars) habitats. (b) For individuals changing their breeding sites, the accumulated proportion of dispersing urban (grey line) and rural (black line) individuals as a funciton of distance is also shown. The maximum dispersal distance observed is indicated by a point (grey and black, for urban and rural birds respectively). Vertical dashed lines show mean distances for urban (grey line) and rural (black line) birds.”

should read:

“(a) Proportion of burrowing owls showing site fidelity (1) or changing their breeding sites between successive years (0) in rural (grey bars) and urban (black bars) habitats. (b) For individuals changing their breeding sites, the accumulated proportion of dispersing urban (black line) and rural (grey line) individuals as a function of distance is also shown. The maximum dispersal distance observed is indicated by a point (black and grey, for urban and rural birds respectively). Vertical dashed lines show mean distances for urban (black line) and rural (grey line) birds.”

and in the Legend of Figure 2, where:

“(a) Factors affecting site fidelity among rural and urban burrowing owls (estimate ± 95% CI). Site fidelity was negatively related to individual behaviour (measured as flight initiation distances, FID) among rural individuals (b) while it was negatively related to conspecific density (measured as aggregation) among urban ones. (c) Lines (black: rural, grey: urban) show the probability of remaining in the same breeding site for individuals with different FID and living at different conspecific densities. Dots (black: rural, white: urban) show predicted values.”

should read

“(a) Factors affecting site fidelity among rural and urban burrowing owls (estimate ± 95% CI) (based on SP2). Site fidelity was negatively related to individual behaviour (measured as flight initiation distances, FID) among rural individuals (b) while it was negatively related to conspecific density (measured as aggregation) among urban ones. (c) Lines (black: urban, grey: rural) show the probability of remaining in the same breeding site for individuals with different FID and living at different conspecific densities. Dots (black: urban, white: rural) show predicted values.”

