# Author Correction: Personality-dependent breeding dispersal in rural but not urban burrowing owls

**DOI:** 10.1038/s41598-023-32912-x

**Published:** 2023-04-12

**Authors:** Álvaro Luna, Antonio Palma, Ana Sanz-Aguilar, José L. Tella, Martina Carrete

**Affiliations:** 1 Department of Conservation Biology, Estación Biológica de Doñana - CSIC, Seville, Spain; 2 Population Ecology Group, Institut Mediterrani d’Estudis Avançats IMEDEA (CSIC-UIB), Majorca, Spain; 3 Department of Physical, Chemical and Natural Systems, Universidad Pablo de Olavide, Seville, Spain

Correction to: *Scientific Reports* 10.1038/s41598-019-39251-w, published online 27 February 2019

The original version of this Article contains errors in the Acknowledgements section.

“We thank Sergio Zalba (Universidad Nacional del Sur, Argentina) for his continuous support, the volunteers, technicians and researchers who have contributed to this project over the years, and the neighbours of Bahia Blanca for their hospitality. The project was funded by Projects CGL2012-31888 and CGL2015-71378-P from MINECO (Spain) and Fundación Repsol. A.L. and A.P. were supported by La Caixa-Severo Ochoa International PhD Program (2015 and 2014 respectively).”

should read:

“This work was supported by CGL2015-71378 MINECO/FEDER, UE, Canal Sur TV, Fundación Repsol, Project CGL2012-31888 MITECO, and COOPA20049 from CSIC (Spain). A.L. and A.P. were supported by La Caixa-Severo Ochoa International PhD Program (2015 and 2014 respectively). We thank Sergio Zalba (Universidad Nacional del Sur, Argentina) for his continuous support, the volunteers, technicians and researchers who have contributed to this project over the years, and the neighbours of Bahia Blanca for their hospitality.”

