# Supplementary material for: Personality-dependent breeding dispersal in rural but not urban burrowing owls
Source: Sci Rep. 2019 Feb 27;9:2886. doi: 10.1038/s41598-019-39251-w (PMC6393437; doi:10.1038/s41598-019-39251-w)
Supplement: Supplementary file 1 — Supplementary_Info [file 41598_2019_39251_MOESM1_ESM.pdf]

SUPPLEMENTARY MATERIAL

Personality-dependent breeding dispersal in rural but not urban burrowing owls

Álvaro Luna, Antonio Palma, Ana Sanz-Aguilar, José L. Tella & Martina Carrete

1. Models

Table S1. Alternative models (ΔAICc < 6) obtained to assess the relative importance of individual's traits (age, sex and behaviour, measured as FID), previous breeding experience (breeding success, productivity and predation in the previous year *t-1*) and conspecific density on the dispersal pattern (site fidelity and dispersal distances) of rural and urban (habitat) burrowing owls *Athene cunicularia*. Models are ranked using the Akaike Information Criterion corrected for small sample sizes (AICc).

**Site fidelity**	**df**	**AICc**	**ΔAICc**	**weight**
Habitat*Aggregation, Habitat*Sex, Habitat*FID, Productivity(t-1)	10	194.73	0.00	0.18
Habitat*Sex, Habitat*FID, Habitat*Productivity(t-1)	10	195.61	0.88	0.12
Habitat*Aggregation, Habitat*Sex, Habitat*FID, Breeding success(t-1)	10	196.08	1.36	0.09
Aggregation, Habitat*Sex, Habitat*FID, Productivity(t-1)	10	196.25	1.52	0.09
Habitat*Aggregation, Habitat*Sex, Habitat*FID	9	197.35	2.62	0.05
Habitat*Sex, Habitat*FID, Habitat*Breeding success(t-1)	10	197.49	2.77	0.05
Aggregation, Habitat*Sex, Habitat*FID, Breeding success(t-1)	10	197.80	3.08	0.04
Habitat*Aggregation, Habitat*Sex, Habitat*FID, Predation(t-1)	10	198.06	3.33	0.03
Habitat*Sex, Habitat*FID, Habitat*Predation(t-1)	10	199.29	4.56	0.02
Aggregation, Habitat*Sex, Habitat*FID	9	199.53	4.80	0.02
Aggregation, Habitat*Sex, Habitat*FID, Predation(t-1)	10	200.05	5.33	0.01
**Dispersal distance**	**df**	**AICc**	**ΔAICc**	**weight**
Null	4	66.62	0.00	0.26

Habitat	5	67.22	0.60	0.19
Habitat*Breeding success(t-1)	7	67.78	1.16	0.15
Habitat*Breeding success(t-1)	7	67.78	1.16	0.15
Predation(t-1)	5	70.58	3.96	0.04
Sex	5	70.61	3.99	0.04
Habitat, Sex	6	70.97	4.35	0.03
Habitat, Predation(t-1)	6	71.27	4.65	0.03
Breeding success(t-1)	5	71.43	4.81	0.02
Habitat, Breeding success(t-1)	6	72.10	5.48	0.02
Sex, Habitat*Breeding success(t-1)	8	72.12	5.50	0.02

Table S2. Relative importance of an individual's traits (sex and behaviour, measured as FID), previous breeding experience (breeding success, productivity and predation in the previous year *t-1*) and conspecific density on the dispersal pattern (site fidelity and dispersal distances) of rural and urban (habitat) burrowing owls *Athene cunicularia*. Estimates and 95% confidence intervals (2.5% and 97.5%) were assessed after model averaging. We considered that a given variable has no, weak or strong support when the 95% confidence interval strongly overlapped zero, barely overlapped zero, or did not overlap zero (in bold), respectively. Models were run using all individuals of unknown age, as age has not received statistical support (see Table 1 and S1). Models shown are those used for model averaging (ΔBIC ≤ 6).

**Site fidelity**	**k**	**AICc**	**ΔAICc**	**weight**
Aggregation+ Habitat*FID, Predation(t-1)	7	1062.36	0.00	0.03
Habitat*FID	5	1062.44	0.08	0.03
Habitat*FID, Predation(t-1)	6	1062.44	0.08	0.03
Aggregation+ Habitat*FID	6	1062.53	0.17	0.03
Sex, Habitat*FID, Predation(t-1)	7	1062.59	0.23	0.03
Aggregation+ Sex, Habitat*FID, Predation(t-1)	8	1062.59	0.23	0.03
Sex, Habitat*FID	6	1062.64	0.28	0.03
Aggregation+ Sex, Habitat*FID	7	1062.81	0.45	0.03
Habitat*Aggregation+ FID	6	1063.10	0.74	0.02
Habitat*Aggregation+ FID, Predation(t-1)	7	1063.19	0.83	0.02
Habitat*Aggregation+ Sex, FID	7	1063.52	1.16	0.02
Habitat*Aggregation+ Sex, FID, Predation(t-1)	8	1063.57	1.21	0.02
Habitat, Aggregation+ FID	6	1063.79	1.43	0.02
Habitat, Aggregation+ FID, Predation(t-1)	7	1063.81	1.45	0.02
Aggregation+ Habitat*FID, Habitat*Predation(t-1)	9	1064.27	1.91	0.01
Aggregation+ Habitat*Sex, Habitat*FID, Predation(t-1)	10	1064.29	1.93	0.01
Aggregation+ FID, Predation(t-1)	6	1064.30	1.94	0.01
Habitat*FID, Breeding success(t-1)	6	1064.31	1.95	0.01
FID, Predation(t-1)	5	1064.31	1.95	0.01
Aggregation+ Habitat*Sex, Habitat*FID	9	1064.32	1.96	0.01
Habitat, Aggregation+ Sex, FID, Predation(t-1)	8	1064.38	2.02	0.01

**Variables**	**Estimate**	**2.5%**	**97.5%**
**Aggregation**	**-0.01**	**-0.03**	**0**
Predation(t-1)	-0.43	-1.15	0.29
**FID*Habitat(rural)**	**-0.02**	**-0.03**	**0**
FID*Habitat(urban)	0.00	-0.01	0.02
Sex(female)	-0.27	-0.76	0.22
**FID**	**-0.01**	**-0.02**	**0**
Aggregation*Habitat(rural)	0.01	-0.02	0.04
**Aggregation*Habitat(urban)**	**-0.02**	**-0.04**	**0**
Habitat(urban)	0.36	-0.25	0.96
Habitat(urban)*Predation(t-1)	0.01	-1.38	1.4
Habitat(urban)*Sex(female)	0.28	-0.54	1.09
Breeding success(t-1)	-0.09	-0.53	0.36
Productivity(t-1)	-0.01	-0.11	0.08
Habitatrural*Productivity(t-1)	-0.07	-0.27	0.12
Habitat(urban)*Productivity(t-1)	0.01	-0.10	0.11
Breeding success(t-1)*Habitat(urban)	0.15	-0.73	1.03

Habitat*FID, Productivity(t-1)	6	1064.39	2.03	0.01
FID	4	1064.39	2.03	0.01
Aggregation+ Habitat*FID, Breeding success(t-1)	7	1064.40	2.04	0.01
Habitat, Aggregation+ Sex, FID	7	1064.41	2.05	0.01
Aggregation+ Habitat*FID, Productivity(t-1)	7	1064.48	2.12	0.01
Sex, Habitat*FID, Breeding success(t-1)	7	1064.51	2.15	0.01
Aggregation+ FID	5	1064.55	2.19	0.01
Sex, Habitat*FID, Productivity(t-1)	7	1064.60	2.24	0.01
Habitat*Aggregation+ Habitat*Sex, Habitat*FID	10	1064.63	2.27	0.01
Aggregation+ Sex, Habitat*FID, Breeding success(t-1)	8	1064.69	2.33	0.01
Sex, FID, Predation(t-1)	6	1064.76	2.40	0.01
Aggregation+ Sex, Habitat*FID, Productivity(t-1)	8	1064.77	2.41	0.01
Habitat, FID	5	1064.77	2.41	0.01
Habitat*Aggregation+ Habitat*Sex, Habitat*FID, Predation(t-1)	11	1064.79	2.43	0.01
Aggregation+ Sex, FID, Predation(t-1)	7	1064.82	2.46	0.01
Sex, FID	5	1064.89	2.53	0.01
Habitat, FID, Predation(t-1)	6	1064.90	2.54	0.01
Habitat*Aggregation+ Habitat*FID, Habitat*Predation(t-1)	10	1064.97	2.61	0.01
Habitat*Aggregation+ FID, Breeding success(t-1)	7	1064.99	2.63	0.01
Habitat*Aggregation+ FID, Productivity(t-1)	7	1065.04	2.68	0.01
Aggregation+ Sex, FID	6	1065.13	2.77	0.01
Habitat, Sex, FID	6	1065.28	2.92	0.01
Habitat, Sex, FID, Predation(t-1)	7	1065.36	3.00	0.01
Habitat*Aggregation+ Sex, FID, Breeding success(t-1)	8	1065.41	3.05	0.01
Habitat*Aggregation+ Sex, FID, Productivity(t-1)	8	1065.46	3.10	0.01
Habitat, Sex	5	1065.50	3.14	0.01
Sex, Habitat*FID, Habitat*Predation(t-1)	9	1065.50	3.14	0.01
Habitat, Aggregation+ Sex	6	1065.51	3.15	0.01
Habitat	4	1065.57	3.21	0.01
Habitat, Aggregation	5	1065.58	3.22	0.01

Habitat, Aggregation+ FID, Breeding success(t-1)	7	1065.60	3.24	0.01
Habitat*Aggregation+ Habitat*FID, Habitat*Productivity(t-1)	9	1065.65	3.29	0.01
Habitat, Aggregation+ FID, Productivity(t-1)	7	1065.70	3.34	0.01
Aggregation+ Habitat*Sex, FID, Predation(t-1)	9	1065.73	3.37	0.01
Habitat, Aggregation+ Sex, Predation(t-1)	7	1065.80	3.44	0.01
Aggregation+ FID, Habitat*Predation(t-1)	8	1065.81	3.45	0.01
Habitat, Sex, Predation(t-1)	6	1065.83	3.47	0.01
Aggregation+ Habitat*Sex, FID	8	1065.84	3.48	0.01
Aggregation+ Habitat*FID, Habitat*Breeding success(t-1)	9	1065.92	3.56	0.01
Habitat, Aggregation+ Predation(t-1)	6	1065.96	3.60	0.01
Habitat, Predation(t-1)	5	1065.99	3.63	0.01
Aggregation+ Habitat*FID, Habitat*Productivity(t-1)	8	1066.00	3.64	0.01
Sex, Habitat*FID, Habitat*Productivity(t-1)	8	1066.04	3.68	0.01
Aggregation+ Habitat*Sex, Habitat*FID, Breeding success(t-1)	10	1066.12	3.76	0.01
Habitat, Aggregation+ Sex, FID, Breeding success(t-1)	8	1066.22	3.86	0.00
Aggregation+ Habitat*Sex, Habitat*FID, Productivity(t-1)	10	1066.24	3.88	0.00
FID, Breeding success(t-1)	5	1066.27	3.91	0.00
Habitat, Aggregation+ Sex, FID, Productivity(t-1)	8	1066.32	3.96	0.00
FID, Productivity(t-1)	5	1066.34	3.98	0.00
Aggregation+ FID, Breeding success(t-1)	6	1066.44	4.08	0.00
Habitat*Aggregation+ Habitat*Sex, Habitat*FID, Breeding success(t-1)	11	1066.44	4.08	0.00
Habitat*Aggregation+ Habitat*FID, Habitat*Breeding success(t-1)	10	1066.45	4.09	0.00
Aggregation+ FID, Productivity(t-1)	6	1066.50	4.14	0.00
Habitat*Aggregation+ Habitat*Sex, Habitat*FID, Productivity(t-1)	11	1066.55	4.19	0.00
Habitat*Sex	6	1066.55	4.19	0.00
Aggregation+ Habitat*Sex	7	1066.59	4.23	0.00
Habitat, FID, Breeding success(t-1)	6	1066.59	4.23	0.00
Habitat*Sex, FID	7	1066.63	4.26	0.00
Habitat*Sex, FID, Predation(t-1)	8	1066.63	4.27	0.00
Habitat, FID, Productivity(t-1)	6	1066.69	4.33	0.00

Aggregation+ Habitat*Sex, Predation(t-1)	8	1066.77	4.41	0.00
Habitat*Sex, Predation(t-1)	7	1066.78	4.42	0.00
Sex, FID, Breeding success(t-1)	6	1066.78	4.42	0.00
Sex, FID, Productivity(t-1)	6	1066.85	4.49	0.00
FID, Habitat*Predation(t-1)	7	1066.89	4.53	0.00
Habitat*Aggregation+ Sex	6	1066.89	4.53	0.00
Aggregation+ Sex, FID, Breeding success(t-1)	7	1067.02	4.66	0.00
Sex, Habitat*FID, Habitat*Breeding success(t-1)	9	1067.07	4.71	0.00
Aggregation+ Sex, FID, Productivity(t-1)	7	1067.08	4.72	0.00
Habitat, Sex, FID, Breeding success(t-1)	7	1067.11	4.75	0.00
Habitat*Sex, Habitat*FID, Habitat*Predation(t-1)	10	1067.16	4.80	0.00
Habitat, Sex, FID, Productivity(t-1)	7	1067.21	4.85	0.00
Habitat, Aggregation+ Sex, Breeding success(t-1)	7	1067.28	4.92	0.00
Habitat*Aggregation+ Sex, Predation(t-1)	7	1067.28	4.92	0.00
Habitat, Sex, Breeding success(t-1)	6	1067.29	4.93	0.00
Habitat, Aggregation+ Breeding success(t-1)	6	1067.34	4.98	0.00
Habitat, Breeding success(t-1)	5	1067.35	4.99	0.00
Sex, FID, Habitat*Predation(t-1)	8	1067.36	5.00	0.00
Habitat, Aggregation+ Sex, Productivity(t-1)	7	1067.43	5.07	0.00
Habitat, Sex, Productivity(t-1)	6	1067.43	5.07	0.00
Aggregation+ FID, Habitat*Breeding success(t-1)	8	1067.47	5.11	0.00
Habitat, Aggregation+ Productivity(t-1)	6	1067.49	5.13	0.00
Habitat, Productivity(t-1)	5	1067.50	5.14	0.00
Aggregation+ Habitat*Sex, FID, Breeding success(t-1)	9	1067.64	5.28	0.00
Habitat*Aggregation+ Habitat*Sex, Habitat*Predation(t-1)	10	1067.65	5.29	0.00
Habitat*Aggregation	5	1067.72	5.36	0.00
Aggregation+ Habitat*Sex, FID, Productivity(t-1)	9	1067.75	5.39	0.00
Aggregation+ Sex, Habitat*Predation(t-1)	8	1067.79	5.43	0.00
Sex, Habitat*Predation(t-1)	7	1067.80	5.44	0.00
FID, Habitat*Productivity(t-1)	6	1067.87	5.51	0.00

Habitat*Predation(t-1)	6	1067.94	5.58	0.00				
Aggregation, Habitat*Predation(t-1)	7	1067.94	5.58	0.00				
Aggregation+ FID, Habitat*Productivity(t-1)	7	1068.08	5.72	0.00				
Habitat*Aggregation+ Predation(t-1)	6	1068.20	5.84	0.00				
Habitat*Sex, Habitat*FID, Habitat*Productivity(t-1)	10	1068.31	5.95	0.00				
Habitat*Sex, Breeding success(t-1)	7	1068.32	5.96	0.00				
Aggregation+ Habitat*Sex, Breeding success(t-1)	8	1068.34	5.98	0.00				
**Dispersal distance**	**k**	**AICc**	**ΔAICc**	**weight**	**Variables**	**Estimate**	**2.5%**	**97.5%**
Habitat, Breeding success(t-1)	6	447.20	0.00	0.32	**Habitat(urban)**	**-0.28**	**-0.50**	**-0.06**
Habitat*Breeding success(t-1)	7	448.10	0.90	0.21	**Breeding success(t-1)**	**-0.25**	**-0.46**	**-0.04**
Habitat*Breeding success(t-1)	7	448.10	0.90	0.21	Breeding success(t-1)*Habitat(urban)	0.21	-0.01	0.43
Habitat, Sex, Breeding success(t-1)	7	449.26	2.07	0.12	**Sex(female)**	**0.09**	**0**	**0.18**
Sex, Habitat*Breeding success(t-1)	8	450.36	3.16	0.07	**Productivity(t-1)**	**-0.04**	**-0.06**	**-0.01**
Habitat	5	452.80	5.61	0.02				
Habitat, Productivity(t-1)	6	453.07	5.88	0.02				

Table S3. Relative importance of an individual's traits (sex and behaviour, measured as FID), previous breeding experience (breeding success, productivity and predation in the previous year *t-1*) and conspecific density on the dispersal pattern (site fidelity and dispersal distances) of rural burrowing owls *Athene cunicularia* with FID within the range of urban ones (5-87m). Estimates and 95% confidence intervals (2.5% and 97.5%) were assessed after model averaging. We considered that a given variable has no, weak or strong support when the 95% confidence interval strongly overlapped zero, barely overlapped zero, or did not overlap zero (in bold), respectively. Models shown are those used for model averaging (ΔAICc ≤ 6).

**Site fidelity**	**df**	**BIC**	**ΔBIC**	**weight**
FID	4	198.15	0.00	0.14
FID, Productivity(t-1)	5	199.35	1.20	0.08
FID, Breeding success(t-1)	5	199.39	1.24	0.07
Sex, FID	5	199.52	1.38	0.07
FID, Predation(t-1)	5	199.62	1.47	0.07
Aggregation, FID	5	199.84	1.70	0.06
Sex, FID, Breeding success(t-1)	6	200.71	2.56	0.04
Sex, FID, Productivity(t-1)	6	200.71	2.57	0.04
Sex, FID, Predation(t-1)	6	200.89	2.74	0.04
Aggregation, FID , Productivity(t-1)	6	201.03	2.88	0.03
Aggregation, FID , Breeding success(t-1)	6	201.07	2.92	0.03
Aggregation, Sex, FID	6	201.15	3.00	0.03
Null	3	201.16	3.01	0.03
Aggregation, FID , Predation(t-1)	6	201.42	3.27	0.03
Sex	4	201.45	3.30	0.03
Aggregation, Sex, FID, Breeding success(t-1)	7	202.32	4.17	0.02
Aggregation, Sex, FID, Productivity(t-1)	7	202.33	4.18	0.02
Breeding success(t-1)	4	202.34	4.19	0.02
Productivity(t-1)	4	202.38	4.24	0.02

**Variables**	**Estimate**	**2.5%**	**97.5%**
**FID**	**-0.38**	**-0.74**	**-0.01**
Productivity(t-1)	-0.16	-0.5	0.19
Breeding success(t-1)	-0.35	-1.11	0.42
Sex(female)	-0.34	-1.07	0.38
Predation(t-1)	-0.42	-1.57	0.73
Aggregation	0.11	-0.24	0.45

Aggregation	4	202.52	4.37	0.02				
Sex, Breeding success(t-1)	5	202.56	4.41	0.02				
Predation(t-1)	4	202.57	4.43	0.02				
Aggregation, Sex, FID , Predation(t-1)	7	202.63	4.49	0.01				
Sex, Productivity(t-1)	5	202.65	4.51	0.01				
Sex, Predation(t-1)	5	202.70	4.55	0.01				
Aggregation, Sex	5	202.73	4.59	0.01				
Aggregation, Breeding success(t-1)	5	203.68	5.53	0.01				
Aggregation, Productivity(t-1)	5	203.73	5.58	0.01				
Aggregation, Sex, Breeding success(t-1)	6	203.83	5.68	0.01				
Aggregation, Sex, Productivity(t-1)	6	203.93	5.78	0.01				
Aggregation, Predation(t-1)	5	204.09	5.94	0.01				
**Dispersal distance**	**df**	**BIC**	**ΔBIC**	**weight**	**Variables**	**Estimate**	**2.5%**	**97.5%**
Breeding success(t-1)	5	101.85	0.00	0.82	**Breeding success(t-1)**	**-0.40**	**-0.60**	**-0.20**
Sex, Breeding success(t-1)	6	106.52	4.67	0.08	Sex(females)	0.05	-0.14	0.23

2. Estimating personality-related resighting rates

We have analyzed capture-recapture data on breeders for which FID is available. First, we have modeled survival by keeping recapture as general as possible (Table S4). Then, once the structure of survival that minimizes AIC was selected (i.e. a model including temporal variation but not differences between habitats or sexes), we have modeled recapture probabilities (Table S5). The best model indicates a sex effect on recapture probabilities, with higher recapture probabilities for males (Table S6). Moreover, the second best model indicates that habitat effects in interaction with sex could be a good predictor of recapture probabilities but not habitat per se (Table S5). In fact, females at rural areas show the lowest recapture probabilities (Table S7; it is important to mention this "lower" recapture probability is very high).

Table S4. Modelling the effects of time (t), habitat (HAB) and sex (SEX) on survival. k: number of parameters estimated by the model.

MODEL	SURVIVAL	RECAPTURE	AICc	ΔAICc	K	Deviance
1	TIME	SEX*HAB*t	1380.0701	0	38	1301.2036
2	HAB*TIME	SEX*HAB*t	1380.6167	0.5466	45	1286.5855
3	HAB	SEX*HAB*t	1383.0726	3.0025	33	1314.9128
4	SEX*HAB	SEX*HAB*t	1387.1559	7.0858	35	1314.7258
5	CONSTANT	SEX*HAB*t	1387.9109	7.8408	32	1321.8801
6	SEX*t	SEX*HAB*t	1388.7391	8.669	45	1294.7079
7	SEX	SEX*HAB*t	1389.882	9.8119	33	1321.7222
8	SEX*HAB*t	SEX*HAB*t	1400.964	20.8939	58	1278.2145

Table S5. Modelling the effects of time (t), habitat (HAB) and sex (SEX) on recapture using a time-dependent survival structure (see Table S4). k: number of parameters estimated by the model.

MODEL	SURVIVAL	RECAPTURE	AICc	ΔAICc	k	Deviance
1	TIME	SEX	1351.0634	0	10	1330.8562
2	TIME	SEX*HAB	1353.258	2.1946	12	1328.9637
3	TIME	CONSTANT	1356.3104	5.247	9	1338.1411
4	TIME	HAB	1357.9279	6.8645	10	1337.7207
5	TIME	TIME	1359.2953	8.2319	15	1328.8412
6	TIME	SEX*t	1360.1389	9.0755	23	1313.0865
7	TIME	HAB*TIME	1367.5309	16.4675	23	1320.4785
8	TIME	SEX*HAB*t	1380.0701	29.0067	38	1301.2036

Table S6. Estimates and 95% confidence intervals (LCI: 2.5% and UCI: 97.5%) of recapture probabilities based on the model SURVIVAL(t), RECAPTURE(sex) (Model 1, Table S5).

Recapture	Estimate	SE	LCI	UCI
Males	0.976	0.009	0.949	0.989
Females	0.928	0.015	0.892	0.953

Table S7. Estimates and 95% confidence intervals (LCI: 2.5% and UCI: 97.5%) of recapture probabilities based on the model SURVIVAL(t), RECAPTURE(sex*habitat) (Model 2, Table S5).

Recapture	Estimate	SE	LCI	UCI
Males Urban	0.972	0.012	0.935	0.988
Females Urban	0.940	0.017	0.897	0.965
Males Rural	0.987	0.013	0.912	0.998
Females Rural	0.900	0.032	0.817	0.948

Regarding the potential effects of FID on recapture rates, we did not find any relationship (Table S8 and S9). All the 95%CI of the beta estimates of FID included zero, indicating a lack of significant effects of individual's personality on recapture probabilities (Table S9).

Table S8. Testing the effect of an individual's personality (FID), sex (SEX) and habitat (HAB) on recapture probabilities using a time-dependent survival structure (see Table S4). k: number of parameters estimated by the model.

Model	SURVIVAL	RECAPTURE	AICc	ΔAICc	k	Deviance
1	TIME	SEX	1351.0634	0	10	1330.8562
2	TIME	SEX*FID	1354.0572	2.9938	12	1329.7629
3	TIME	FID	1357.0177	5.9543	10	1336.8105
4	TIME	HAB*FID	1360.5552	9.4918	12	1336.2609
5	TIME	SEX*HAB*FID	1360.7111	9.6477	16	1328.1959

Table S9. Estimates and 95% confidence intervals (LCI: 2.5% and UCI: 97.5%) for the effect of individual's personality (FID) on recapture probabilities (based on models presented in Table S8).

Model	Parameter	Estimate	LCI	UCI
2	slope FID males	0.0058866	-0.04005	0.0518231
2	slope FID females	-0.0066979	-0.0180615	0.0046657
3	slope FID	-0.0075621	-0.0187288	0.0036047
4	slope FID urban	0.01136	-0.0408484	0.0635684
4	slope FID rural	-0.0084598	-0.0213536	0.0044341
5	slope FID urban males	0.0058557	-0.098415	0.1101264
5	slope FID urban females	0.0171106	-0.0428763	0.0770976
5	slope FID rural males	-0.0039968	-0.0603889	0.0523953
5	slope FID rural females	-0.0050906	-0.019355	0.0091737
